# Transcription Factor Target Gene Network governs the Logical Abstraction Analysis of the Synthetic Circuit in Leishmaniasis

**DOI:** 10.1038/s41598-018-21840-w

**Published:** 2018-02-22

**Authors:** Milsee Mol, Dipali Kosey, Ramanamurthy Boppana, Shailza Singh

**Affiliations:** National Centre for Cell Science, NCCS Complex, Ganeshkhind, SP Pune University Campus, Pune, 411007 India

## Abstract

With the advent of synthetic biology in medicine many synthetic or engineered proteins have made their way to therapeutics and diagnostics. In this paper, the downstream gene network of CD14-TNF-EGFR pathway in leishmaniasis, a tropical disease, is reconstructed. Network analysis showed that NFkB links the signaling and gene network, used as a point of intervention through a synthetic circuit embedded within the negative autoregulatory feedback loop. A chimeric protein kinase C (PKC) is incorporated in the synthetic circuit, under the transcriptional regulation of Lac repressor and IPTG, as an inducer. The chimeric PKC_ζα via IκKb phosphorylation activates NFκB, and modulates the gene expression from an anti-inflammatory to a pro-inflammatory phenotype in *in vitro L. major* infected macrophage model. This is the first ever report of a synthetic device construction in leishmania.

## Introduction

Leishmaniasis is a group of complex disease caused by the protozoan parasite *Leishmania*. The parasite has two morphological forms namely amastigote and promastigote. *Leishmania* promastigotes are engulfed by the dendritic cells (DCs), neutrophils and macrophages when injected into the mammalian host by an infected sandfly. The infected macrophages may produce interleukin (IL)–12 that activates the natural killer (NKs) cells to produce interferon (IFN) -γ activating T helper (Th)–1 cells. Early synthesis of tumour necrosis factor (TNF)-α from macrophages, synergizes with IFN-γ for intracellular killing of *Leishmania* via oxidative mechanisms. While on the other hand *Leishmania* is also capable of subverting the Th-1 response and initiate Th-2 response for disease progression, which is characterized by the early production of IL-4, IL-10, IL-13 and absence of synthesis of IL-12^1–3^.

This observed skewness of macrophages is ultimately the result of modulated gene expression via signalling components by the *Leishmania* parasite. Research has shown that activated phenotype of macrophages are flexible and they can change from one functional phenotype to another in response to the variable microenvironment^4^. Macrophages can sequentially change their functional phenotype in response to changes in *in vitro* cytokine stimulation^5^. This ability of macrophages to reprogram itself can be tapped for application in immune therapeutics i.e. by stimulating them with chemokines or immune stimulants like lipophosphosaccharide (LPS).

Another possible avenue for inducing plasticity of macrophage phenotype could be using synthetic biology approach to rewire signalling with novel molecular functions. It can be achieved by manipulating endogenous pathways or uploading a heterogeneous completely new pathway into the host cell for cell therapy. Engineered proteins are fascinating to be used for rewiring signalling, as they can be targeted against ligand-receptor or protein-protein interactions that comprise the signalling pathways. This work exemplifies the use of a chimeric protein kinase C (PKC) embedded within a negative autoregulatory synthetic circuit for immune modulation via activation of NFκB which could result in phenotypic change of the *Leishmania* infected macrophages. *Leishmania* has the capability of modulating Nuclear factor-κB (NFκB) as well as PKC isoform activity for its safe intracellular survival. PKC isoforms are modulated by either changing the activity of the regulatory domain or the catalytic domain. It is seen that PKC-ζ is modulated by enhancing its affinity towards its substrate in the presence of ceramide which is concomitantly produced during *Leishmania* infection. At the same time, PKC-α catalysis action is dampened by interfering with the binding of co-modulators Ca^2+^ and di-acyl glycerol (DAG)^6–8^. We propose that the domain swapping of the PKC-α and PKC-ζ isoforms to form a chimeric PKC_ζα can be used for changing the substrate docking and catalysis. Therefore a chimeric PKC_ζα composed of the PB1 domain from PKC-ζ and catalytic domain from PKC-α was designed, which may rewire NFkB/RelA by phosphorylation of the IκK-β, which in turn will phosphorylate IκB, and thus, unlock the RelA for nuclear translocation and modulation of gene expression.

The *in silico* designed synthetic circuit is a logical abstraction driven by the systems approach for understanding the signaling of CD14, TNF and EGFR^9^ pathway and their corresponding downstream TF and target gene (TG) network. The reconstructed TFTG network would be the effector system of the activated signal transduction and therefore we begin with its analysis followed by the design and verification of the synthetic circuit and it’s *in vitro* effect on parasite survival.

## Results and Discussion

### Reconstruction of TFTG network and node Selection

Since there are no direct evidence for the transcription factors (TFs) and their corresponding transcription genes (TGs) in leishmaniasis, knowledge from various databases and literature were collected and a graph based analysis was implemented on the reconstructed TFTG network. The whole transcription-factor target gene (TFTG) network was analysed using the network analyser in Cytoscape (V 3.4.0). The TFTG network had 71 genes and 134 TF-TG pairs, from which important TFs and their TGs were selected based on network matrices of closeness centrality (CC) and edge betweeness. CC measures the reciprocal of farness of a node in a network i.e. higher the value of CC more important is the node in the network. The importance of the node is due the fact that higher the CC, faster will be the spread of information to the reachable nodes. In the reconstructed network, NFκB shows the highest CC value (S1), indicating that it is an important node in transmitting signaling information for appropriate gene expression within the CD14, TNF and EGFR network. From the node pair list and the network matrices applied to the TFTG network, it is seen that IL-2, IL-4, IL-5, IL-6, IL-10, IL-12b, iNOS2 and TNF-α can be affected the most, if NFκB is modulated. This *in silico* observation is well supported by the abundant experimental data documented in literature. *Leishmania* virulence factors like protein tyrosine phosphatases (PTPs), glycoinositolphospholipids (GIPLs), lipophosphoglycan (LPG), proteophosphoglycan (PPG), GP 63 and the 11 kDa kinetoplastid membrane protein (KMP-11)^10^ are known to inhibit the activity and nuclear translocation of transcription factors (TFs) such as NFκB, STAT, CREB and AP1^11,12^. The increased intracellular ceramide in *Leishmania* infection inhibits the activation of macrophage NFκB^13–16^. GP63 metalloprotease, one of the important surface markers of *Leishmania*, degrades NFκB subunits within the nuclear compartment^16,17^. *L*. *major* amastigotes induce p50 and cRel containing complexes, which form either p50/p50 homo-dimers or p50/cRel hetero-dimers that translocate to the nucleus altering the cytokine gene expression pattern towards anti-inflammatory state. *Leishmania* are also capable of cleaving NFκB p65/Rela to p35/Rela, which hetero-dimerizes with p50. It is a GP63-dependent cleavage, which is responsible for induction of anti-inflammatory chemokines like IL-4 and IL-10^18^. It is also reported that GP63 from *L*. *mexicana* amastigotes completely degrade the NFκB complex and shuts down LPS-induced IL-12 production by macrophages^19^. This results in the deactivation of the Th-1 arm of the immune response, which is incapable of mounting a cytotoxic effect on the infected macrophages^20^.

### Evolvability optimization of the reconstructed TFTG network

Evolvability of a biological system is defined as the amenability for evolutionary changes. Biological evolvability depends upon how malleable the genotype is to phenotypic mapping that creates morphological diversification. *Leishmania* modulates immune signaling in the infected macrophages and changes the phenotype to M-2, which can be rewired via NFκB to M-1 for parasite resolution. That is, the TFTG network corresponding to the CD14, TNF and EGFR pathway should evolve to a new pro-inflammatory phenotype. Since evolvability is a multi-objective optimization problem, the system under consideration was deciphered by implementing multi objective genetic algorithm (MOGA), using the following objective function.

$$\begin{array}{c}function\,y=TFTG\,(x)\\ f(1)=-{((\exp (1\ast x(1))+abs(1\ast x(2))+2\ast x(3)+abs(2\ast x(4))))}^{2};\,pro-inflammatory\\ f(2)={((\exp (2\ast x(1))+abs(2\ast x(2))+1\ast x(3)+abs(1\ast x(4))))}^{1};\,anti-inflammatory\\ end\end{array}$$

**Figure 1:**
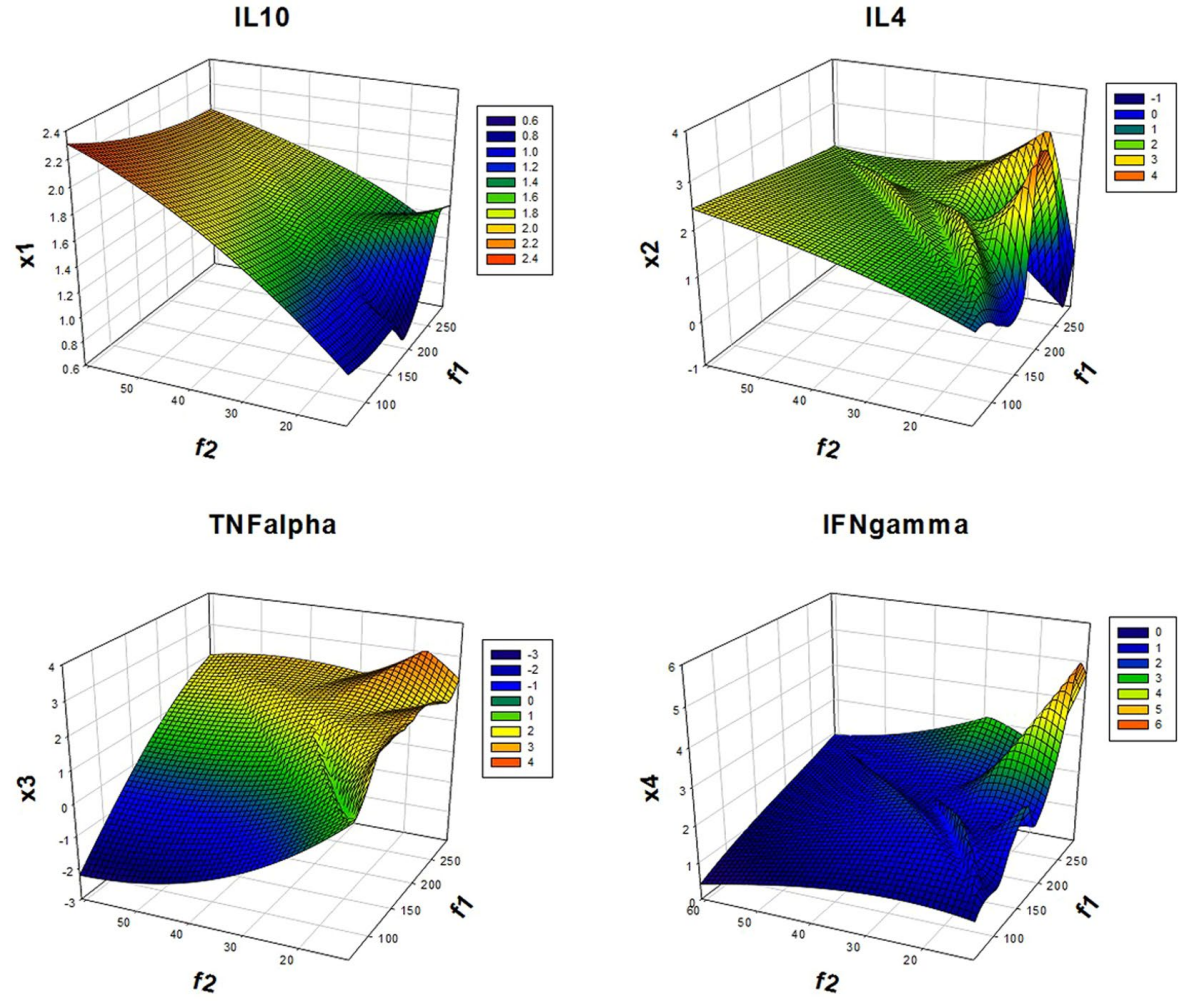
Fitness value V/s decision variable for the anti-inflammatory cytokines IL10 and IL4 and pro-inflammatory cytokines TNFalpha and IFNgamma as evaluated by the f(1) and f(2) objective function.

**Figure 2:**
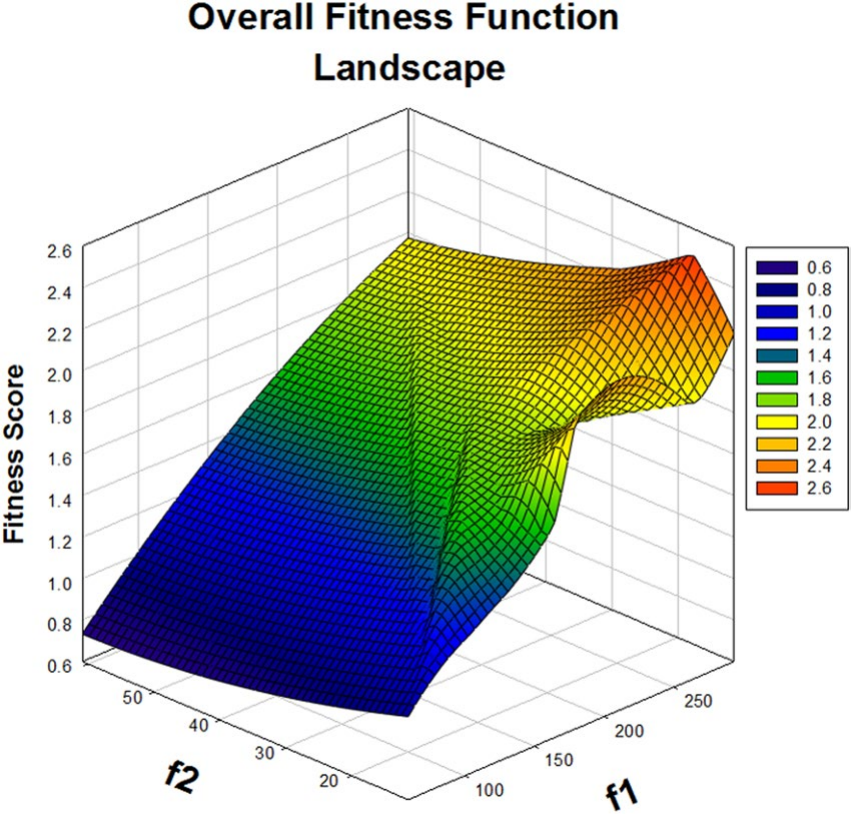
The overall fitness function landscape for the objective function f(1) and f(2) with the score colour scale for these objective functions.

The objective function was solved by the “gamultiobj” solver and shows that the system may evolve to a new phenotype, under the laid constraints. As cited in the materials and methods section, the non-dominated sorting genetic algorithm (NSGA-II) algorithm, is based on the principle of elitism, preserving the ‘elite’ population (S2(a)), through the run of GA; evident in the score histogram (S2(b)). It shows that f(1) is higher compared to f(2) and also the number of individuals i.e. solutions to the given objective function lie more in favour of f(1). The f(1) function defines the pro-inflammatory state, thus it can be said that the system can evolve to a new phenotype, if probed with a synthetic circuit for NFκB activation. The Pareto fronts (S2(c)) for the opposing objective functions have 19 non-dominated solutions that are not discontinuous and the average spread measure (S2(d)) for these solutions is 0.129942. This was the smallest average distance measure achieved with iterative runs of GA of the defined ‘TFTG’ objective function, indicating that the solutions on the Pareto front are evenly distributed. The decision variables embedded in f(1) and f(2) objective functions are cytokines, whose expression levels would be measured in *in vitro* experiments. They are denoted as x1, x2, x3 and x4, representing the cytokines IL-10, IL-4, TNF-α and IFN-γ respectively. The fitness value obtained for the objective function decides the trend that is followed by the decision variable which is depicted in Fig. 1. It shows that x1 and x2 decision variables representing the anti-inflammatory cytokines show a rugged oscillatory behaviour, converging to the f(2) (anti-inflammatory) fitness function. Similarly, x3 and x4 converges towards the f(1) (pro-inflammatory) fitness function. The overall fitness landscape (Fig. 2) for the two objectives shows a convergence to f(1) which is the ultimate goal of the multi-objective optimization in this study. (Note: Convergence here means higher fitness value assigned to the decision variables). The *in silico* prediction favours the f(1) objective, which we have achieved using a synthetic circuit constituting an engineered PKC protein to modulate the activation of NFκB.

### Chimeric PKC_ζα, its homology modeling and Molecular Dynamic (MD) simulation

The sequence of amino acids (S3(a)) of the chimeric PKC_ζα was analyzed by the ProtParam^21^ tool for the physiochemical properties (S3(b)). The PDB templates used for its homology modeling were 1WMH and 4RA4 from *Homo sapiens*. The homology model of chimeric PKC_ζα, shows about 96.5% of amino acid residues in the favored region and 2.6% in the allowed region. Eleven amino acids were in the outlier region and so loop refinement of the structure was done after which the number of outlier amino acids reduced to 4 i.e. 0.9%. This model was used as the starting model for MD simulation and the protein shows stable dynamics over the 15 ns simulation. All the physical parameters viz temperature, volume and pressure were maintained during the 15 ns MD (S4(a)) production run. Root mean square deviation (RMSD) plot (S4(b)) of 15 ns MD simulation showing the mean fluctuation was at 8 Angstrom and that stabilized after 10 ns; while root mean square fluctuation (RMSF) plot (S4(b)) of the 462 amino acids of the chimeric PKC_ζα shows, that, apart from the hinge/loop of the chimeric PKC_ζα all the other amino acids show a mean fluctuation within the 1 Angstrom range. Modelling errors were checked on ProSA-Web which returns the z-score and energy plots for the chimeric PKC_ζα. Z-scores of the chimeric PKC_ζα is well within the z-scores of all the experimentally (X-ray, NMR) determined protein structures in PDB. Similarly, the energy plot shows the local model quality by plotting energies as a function of amino acid sequence at a particular position (S4(c)). The energy values of the modeled PKC_ζα are negative, confirming an error free 3D structure. The Ramachandran plot for the final 3D model is shown in S4(d). The final 3D structure after MD simulation of the chimeric PKC_ζα is shown in S4(e).

### Synthesis, expression and identification of the chimeric PKC_ζα

The artificially synthesized construct was analyzed by restriction digestion (S5(a)) which confirmed the chimeric PKC_ζα insert. The chimeric PKC_ζα was successfully transformed in to BL21 (DE3) and induced for the expression of chimeric PKC_ζα with 1 mM IPTG for 1 hr. The expressed protein was identified as chimeric PKC_ζα by MS analysis (S5(b)) and anti-His probing by western (S5(c)).

### Negative autoregulatory circuit design and its quasipotential landscape

**Figure 3:**
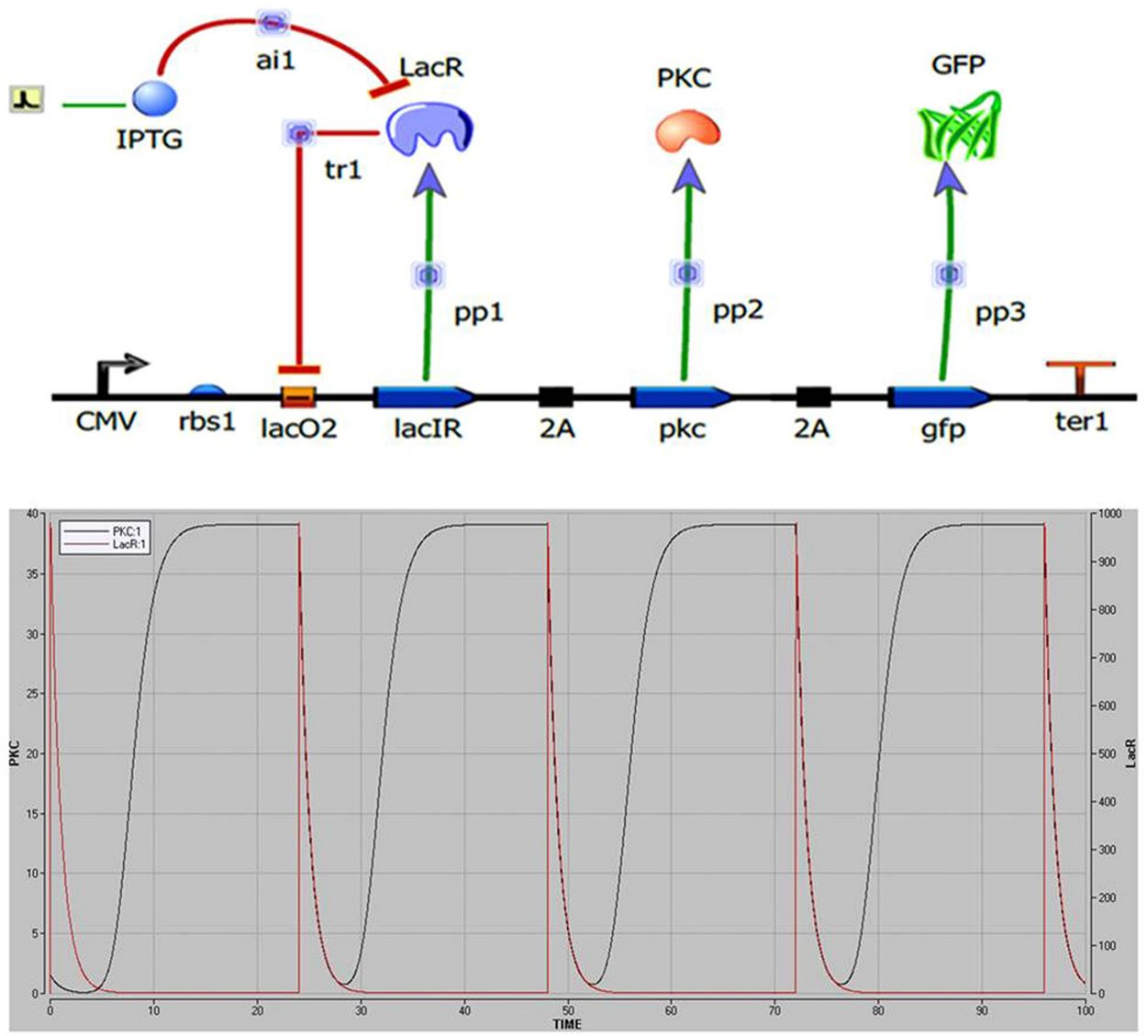
(**a**) Circuit Design; and (**b**) simulation results showing oscillations between the LacR and PKC due to the intermittent pulse of IPTG of 1 mM.

The negative autoregulatory circuit (Fig. 3(a)) *in silico* simulation was done in Berkeley Madonna (v 8.3), using a modified equation inspired from the toggle switch of Gardner *et al*.^22^. The circuit has a single negative feedback loop in the form of LacI repressor protein, whose activity dictates the level of chimeric PKC_ζα in the system. The equations for the synthetic circuit are given in S6(a). The simulation shows the oscillations generated due to impulse of IPTG at 24 hour time point at a concentration of 1 mM (Fig. 3(b)).

**Figure 4:**
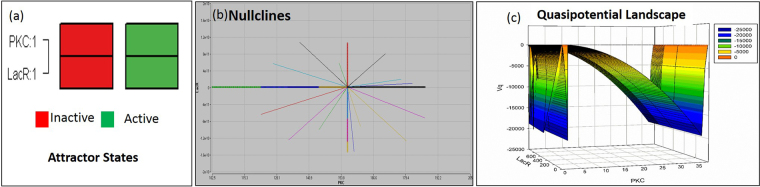
BoolNet Analysis (**a**) Attractor states; (**b**) Nullcline phase plane; (**c**) Quasipotential landscape shows how the system crosses the thermodynamic barriers to move from one state to the other.

The time series data generated in Berkeley Madonna was further analyzed in R BoolNet package, which shows that the system has two attractor states, i.e. either ON or OFF state. Attractor states suggest the stable gene expression pattern of the Boolean network. This system of a single negative autoregulator can oscillate between the two stable states ON and OFF in the presence or absence of the inducer isopropyl β-D-1-thiogalactopyranoside (IPTG), respectively. This helps in tuning the circuit response to the IPTG concentration. The network wiring shows how the components are wired with respect to each other i.e. LacI R regulates itself and the expression of chimeric PKC_ζα (S6(b)). A state transition network depicts the binary representation of the attractors and the transitions between the attractor states. The total number of transition states in the circuit was two, corresponding to the ON and OFF attractor states (Fig. 4(a)). Nullcline for the two ODEs of the synthetic circuit shows an asymptotically stable state i.e. the initial conditions of the system remain close to the initial condition (limiting behavior) and eventually converge to the equilibrium (Fig. 4(b)). For the synthetic circuit, the initial condition of the system is in the OFF state, which is turned ON with the impulse of the inducer IPTG. The system returns to the initial OFF state as the IPTG concentration falls from 1 mM to zero mM and the system is ready for another impulse of IPTG. All the trajectory of the system converge to the center of the nullcline, at a single point indicating that the system can be in either the ON or OFF state and never in between i.e. intermediate state doesn’t exist. The point of intersection of nullclines is the equilibrium point.

The global dynamics of the system can be inferred by assigning potential to the attractor states. Hence the potential landscape states how forces on the system drive the system to the stable states. For the single negative auto regulatory circuit the force that drives the ON and OFF mechanism is the input of IPTG. The quasipotential landscape shows how the system crosses the thermodynamic barriers to move from one state to the other. Moreover, it can be visualized in Fig. 4(c), that the attractors remain in a state of low potential (OFF) and upon addition of inducer, the system moves towards a higher potential state (ON). Valley in the quasipotential landscape correspond to the attractors and peaks to the transition states that exists between the attractor states.

GRENITS^23^ analysis in R is associated to the adequate convergence of the Markov Chain Monte Carlo (MCMC), which is crucial for further analysis of the synthetic circuit model. For the synthetic circuit there is a perfect convergence of the plots for Gamma, B, Mu, Rho and Lambda which signifies the steady state dynamics and also the robustness of the designed synthetic circuit S6(c).

### Transfection and *in vitro* working of the synthetic circuit

**Figure 5:**
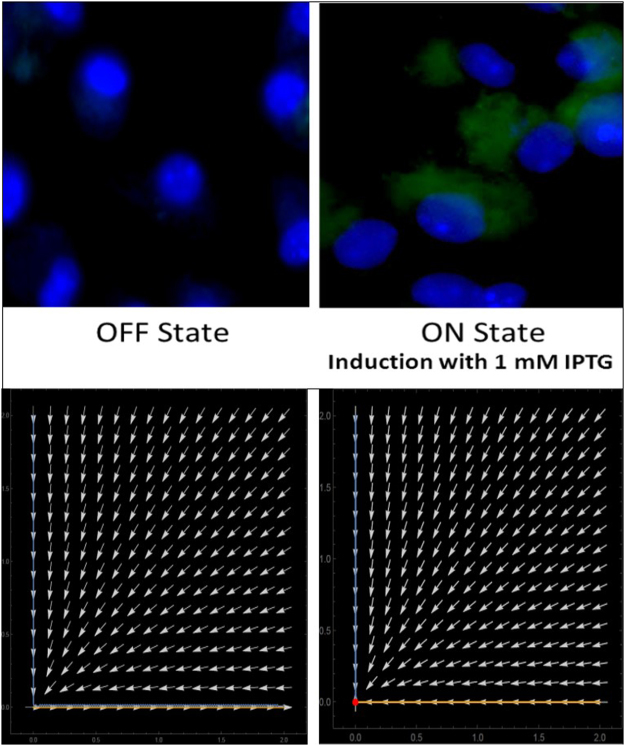
The ON and OFF working of the circuit measured as a function of GFP expression on induction with IPTG: Upper panel shows the OFF (no GFP expression) and ON (GFP expression in the presence of inducer IPTG) state in transfected peritoneal macrophages; Lower panel shows the phase plane diagram explaining the convergence of the system to the ON state.

The chimeric PKC_ζα introduced into the negative autoregulatory circuit was transfected into peritoneal macrophages. The transfection was successful with an efficiency of over 20–30%. The working of the circuit was established with 1 mM IPTG induction for 24 hours and expression of GFP, when the IPTG was withdrawn the circuit was in the OFF state i.e. no GFP production and hence no chimeric PKC_ζα (Fig. 5a). The developed mathematical model has allowed us to predict these changes with varying IPTG concentration (0 and 1 mM). In this way, we have utilized a functional approach rather than biophysical or biochemical detailing, that emphasize the role of feedback loop insertion in our mathematical model. The steady state values of the model and the nullcline obtained reveals a monotonically increasing function with the associated variables. The asymptotically stable attractor is illustrated in the phase plane portrait obtained (Fig. 5b). It is noteworthy to observe a biphasic output with a large initial peak followed by a sustained plateau with smaller amplitude in the quasipotential landscape represented in this paper. This is in agreement with the experimental results. Although changing the model parameters alters the quantitative analyses, the obtained qualitative responses are the same. Thus, it can be said that the absolute values of the obtained results are less significant, therefore the simulation results are qualitatively discussed with the experimental observations. The simulation results actually helped us to analyze the cross-talk points identified in our CD14, TNF and EGFR signaling model system^9^.

**Figure 6:**
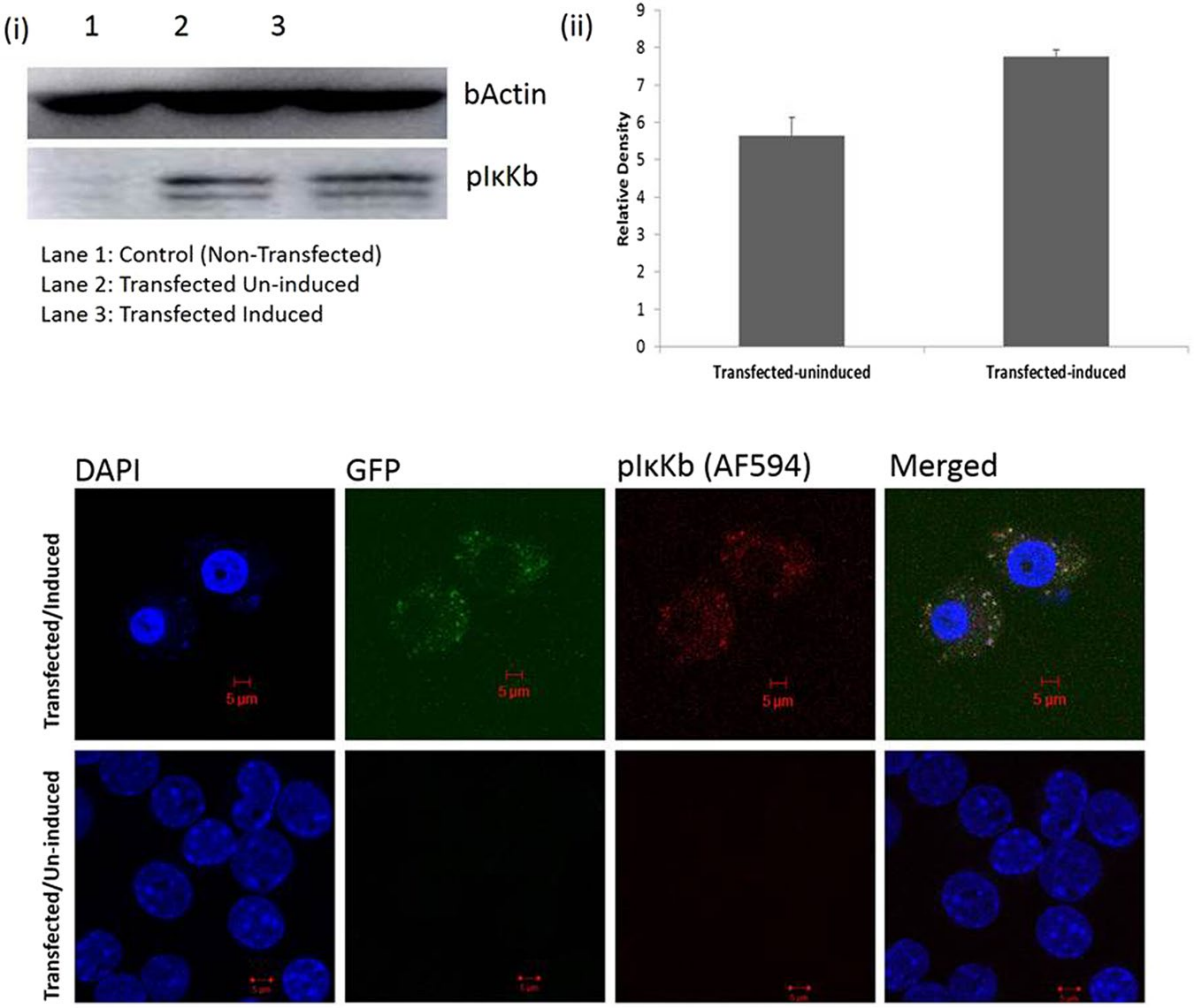
(**a**) Engineered chimeric PKC phosphorylating IκK-β as shown by (i) Western blot and its (ii) densitometry analysis in ImageJ. (**b**) Confocal Image of the transfected peritoneal macrophages showing the phosphorylation of IκK-β on induction with IPTG (1 mM).

Western analysis shows that the engineered chimeric PKC would be responsible for the phosphorylation of IκK-β. As shown in Fig. 6(a), the phosphorylated IκK-β band relative density is higher in the transfected induced lane compared to the transfected non-induced (mock) lane. This phenomenon was also observed in the confocal image (Fig. 6(b)).

### Nitrite and Cytokine analysis

**Figure 7:**
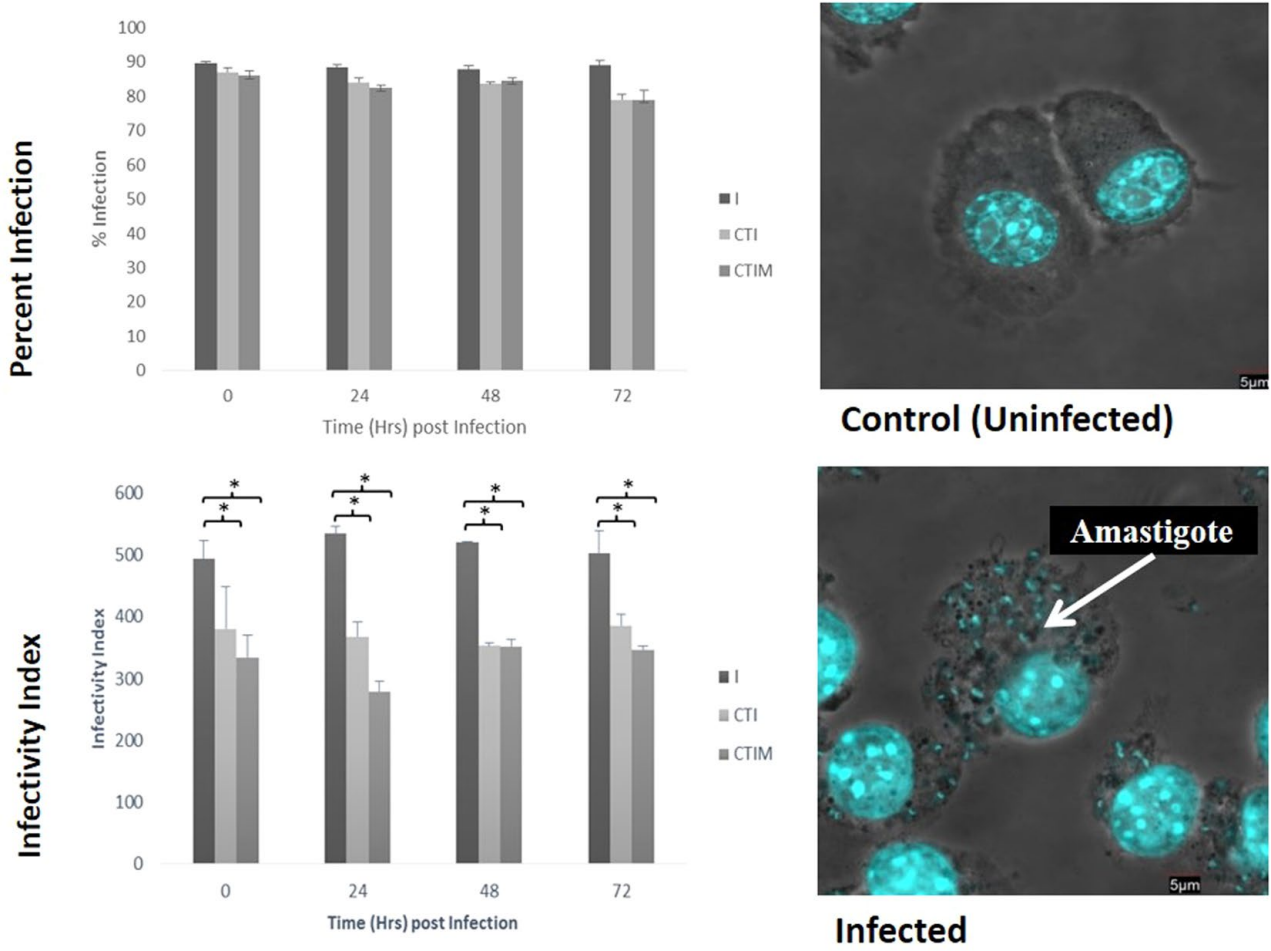
The percent infection and infectivity index of *Leishmania* infected macrophages. The lower panel shows representative figure of uninfected and infected peritoneal macrophages.

The percent infection and infectivity index of the *Leishmania* infected macrophages is shown in Fig. 7. It is seen that in the transfected CTI and CTIM group, the percent infection stays close to 85% when compared to the I group with no significant change. While the infectivity index shows a reduced parasite load in the CTI and CTIM group when compared to I group, suggesting that though there isn’t much change in the percent infection, there is reduction in the parasite load within the macrophages.

**Figure 8:**
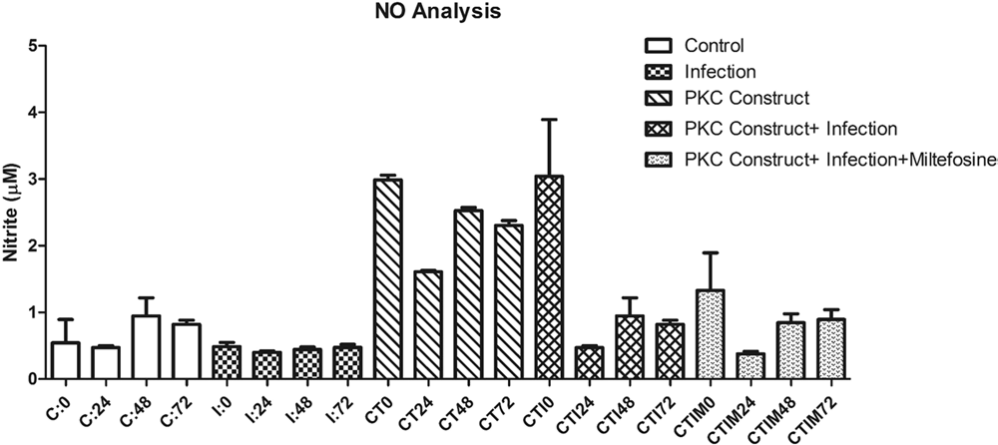
NO profile between the treatment groups.

Nitrite was done for the C, I, LPS, EV, CT, CTI and CTIM groups at the interval of 0, 24, 48 and 72 hrs. The Nitrite estimation for the study groups is shown in Fig. 8. As it can be observed, infection does bring down the nitrite content which is elevated in the CT group. But in the CTI and CTIM group, there is decrease in the nitrite content, albeit not as low as in infection, suggesting that there is some NO production which is the cause for the lower parasite load in these groups. The NO production has not reached the levels of LPS treatment. Since, nitrite is an estimation of iNOS activity in a pro-inflammatory state, it can be said that the chimeric PKC_ζα is showing an effect on the expression of pro-inflammatory cytokine, in this case the nitrite level. This may be causing intracellular killing of the amastigote within the infected macrophages, as is evident in the infectivity index in the CTI and CTIM groups. The EV group did not show any effect on the nitrite levels and remains close to what is measured in C group and LPS was used as a positive control (S10).

**Figure 9:**
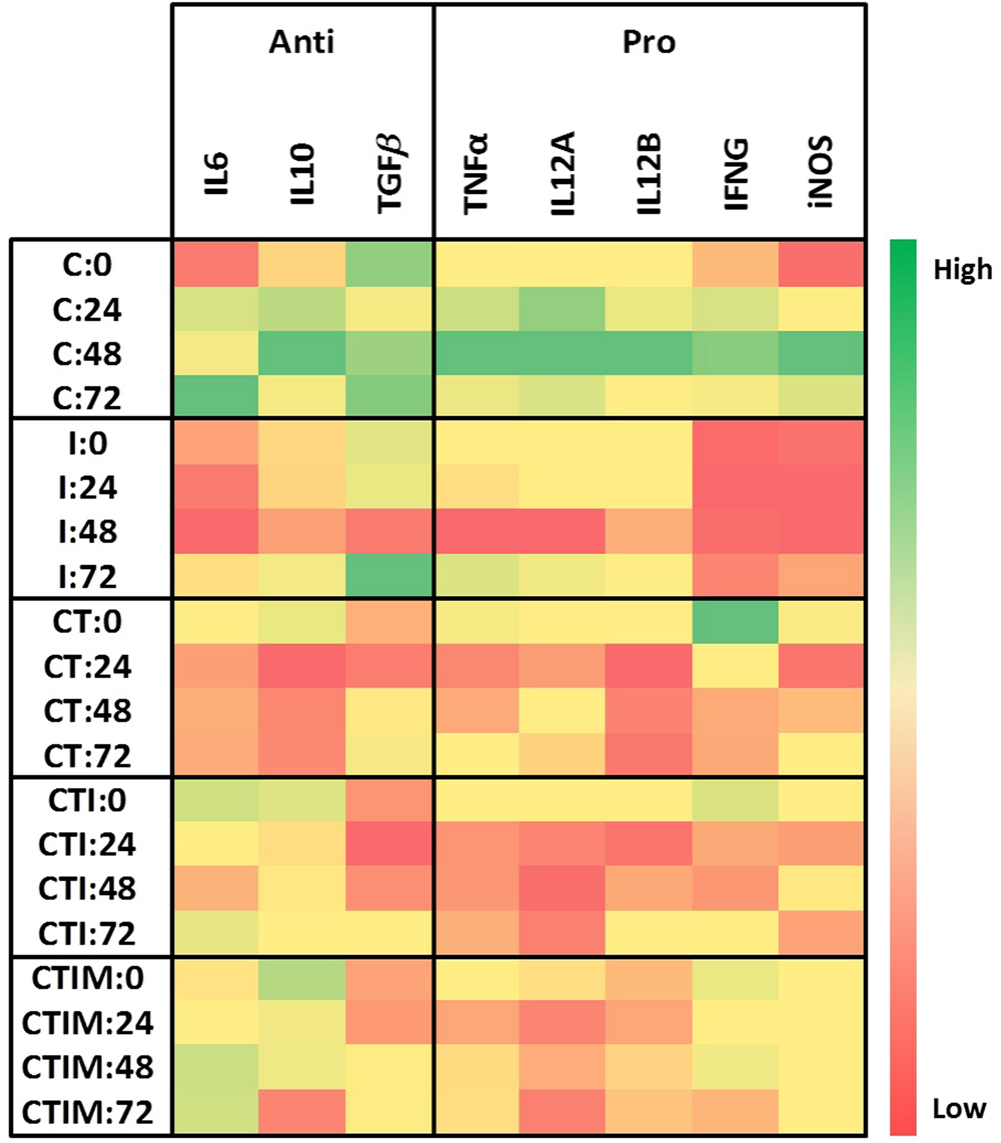
Cytokine analysis by qPCR between the treatment groups compared to the untreated control group.

While the cytokine qPCR analysis for the C, I, CT, CTI and CTIM groups shows a change in the pro-inflammatory and anti-inflammatory cytokines levels between the treatment groups (Fig. 9; Supplementary S9). It was seen that IL-4 cytokine expression level was high in I group, while the other treatment groups i.e. CT, CTI, and CTIM did not have detectable amount of relative mRNA levels. Similarly, there is in fact a shift in the cytokine profile from an anti-inflammatory (TGF-β, IL-10) state to a pro-inflammatory (iNOS, IFN-γ, IL-12B) state in the CTI and CTIM group.

This work thus tries to skew the macrophage phenotype in *Leishmania* infection model system using a simple synthetic gene circuit for NFκB modulation. There have been many instances where NFκB has been modulated for therapeutic effects. Clonidine, an anti-hypertension agent is effectively used as adjunct to anesthesia/sedation and pain management. It has shown to enhance iNOS expression in endotoxin-activated macrophages via induction of NFκB^24^. Since PKC is associated with NFκB activation by a variety of stimuli. Anti-tumor drugs like Vincristine, daunomycin, and paclitaxel have been demonstrated to significantly increase PKC activity, which also resulted in NFκB induction and tumor reduction^25^. Inversely, NFκB has been also implicated in drug resistance in cancer treatments and many of the drugs now are being synthesised targeting the activity of NFκB, e.g. treatment of cells in murine models with genistein, an AKT-NF-κB inhibitor, nullified the increased NFκB activity, sensitized them to cisplatin induced apoptosis^26^. But, no synthetic circuit has been conceived for the activation of NFκB.

Systems and synthetic biology have set their bench mark in the field of cancer and metabolic syndrome for therapeutics and diagnostics. Metabolic syndrome is characterized by the three interlinked and interdependent metabolic disorders of hypertension, hyperglycemia, and obesity^27,28^ which are treated independent of each other. A multifunctional synthetic gene circuit and a combination of drugs can improve the clinical manifestations of this syndrome. Guanabenz is an antihypertensive drug which controls the expression of a fusion hormone glucagon like peptide (GLP)−1-Fc-Leptin, in a dose dependent manner which simultaneously attenuates hypertension by guanabenz, hyperglycaemia by GLP-1 and obesity by Leptin^29^. Another example is a transgene circuit with a dual-promoter was developed capable of discriminating and killing cancer cells. Two different tumor specific promoters drive the expression of chimeric proteins DocS-VP16 and Coh2-GAL4. When both are expressed (AND logic gate) in the presence of a cancer specific signal, they dimerize to form an active PGAL4 promoter transactivation protein that triggers the expression of thymidine kinase (TK). This TK converts the prodrug ganciclovir to the active drug killing the cancer cells^30^. Similarly, T cell reprogramming for immunotherapy in chronic lymphocytic leukemia was achieved by designing a chimeric antigen receptor (CAR) made up of four functional domains: an anti-CD19 single chain fragment, a human CD137-derived co-stimulatory domain, a CD3f signaling domain and a CD8a-derived transmembrane domain. In chronic lymphocyte leukemia cells express the CD19 marker, which is recognized by the CAR expressing transgenic T cells. On recognition, they are activated for proliferation and destruction of the cancer cells^31^. Likewise, antibodies fused to therapeutic proteins can be used for targeted activity, like cytokines fused to antibodies or receptor ligands, can be targeted to cells that express the corresponding antigen or receptor, respectively^32,33^. There are no reports of synergistic application of systems and synthetic biology in the field of leishmaniasis. This work is the first of its kind in the leishmanial infection system. The system guided design of the synthetic circuit, shows a positive effect on the generation of pro-inflammatory mediator NO, TNF-α, IL-12 and IFN-γ. This system may be improved further for a desired modulatory effect, for a cell to cell communication by fine tuning the response of the synthetic circuit in macrophage-T cell co-culture studies.

Interfacing the computational and synthetic biology tools accelerate our understanding of complex biological systems and also our ability to quantitatively engineer cells that may aid as therapeutic device. Cells are basically engineered either to aid in various delivery platforms or to sense a specific molecule(s) that may distinguish the diseased vs. healthy state of the infection model system chosen for the study. The crux, here, lies into having a hierarchical architecture of the functional modules (parts assembled in a way to form an integrated biological system). The system is robust to changes in parameter values as this is a context dependent system we have focused on, while laying the basic principle design of the synthetic signaling circuit.

The study began with a motivation when we identified that a negative auto regulatory module is embedded within a large transcriptional network. Like, electronic circuits are designed so that the connection of new inputs or outputs does not in any way impact the properties of a given module so are the synthetic biological circuits. The promoter binds and sequesters protein that has pleiotropic effects over the entire network. If the total promoter concentration is small compared to the TF-promoter equilibrium constant, sequestration effects are negligible and we do expect the total promoter concentration to be reliable. The promoter concentration ensures the synthetic device component reliability very much like the electronic circuits.

In addition, by designing synthetic systems which are translated and transcribed by completely orthogonal gene expression machinery offers another way of taking advantage of a host cell without interfering with its components. The effects a host plays on the synthetic circuit behaviour should be either minimized or where applicable should be quantified in order to characterize and validate parts/modules so as to minimize crosstalk between an engineered circuit and a host’s machinery. Thus, in the cellular context the designed synthetic circuit has better orthogonality (decoupling) with higher predictability and robustness.

In nutshell, we present paradigms for the design, construction and validation of robust synthetic circuits which may act as an implantable therapeutic device with more scale-ups or reuse of the modules in future.

## Materials and Methods

### Reconstruction of TFTG network and Node selection

There were nine TFs that could be related to *Leishmania* infection corresponding to the previously reconstructed CD14, TNF and EGFR^9^ signaling network namely CREB1, ELK1, ETS1, FOS, JUN, MYC, NFKB, STAT1 AND STAT3. The reconstructed TFTG network consists of possibly all the target genes that are involved in an immune response in leishmaniasis. The TGs of these nine TFs in mice were retrieved from Regulatory Network Repository (http://www.regnetworkweb.org/) and Transcriptional Regulatory Element Database (TRED; http://www.cb.utdallas.edu/cgi-bin/TRED). The association of these TGs with leishmaniasis was done through extensive literature survey and database mining. The curated TG list, thus derived, was also matched to RNA-seq data that dealt with comparison of *L*. *major* infection in peritoneal macrophages at different time points^34^. In total, 71 genes were identified and they were associated with the nine TFs based on information retrieved from RegNetwork and TRED. The TFTG network thus built has a bipartite architecture having 137 TFTG pair list, which was analysed in Cytoscape (V 3.4.0).

### Multi objective optimization and evolvability

The TFTG network optimization was achieved by defining two opposing objective functions; f(1): considers the system associated with anti-inflammatory condition and f(2): considers the system associated with pro-inflammatory condition, given below. The multi-objective optimization^35^ was performed in MATLABs’ Optimization toolbox (7.11.1.866) (The MathWorks Inc.) using the function solver “gamultiobj”.

### Chimeric PKC design

The chimeric PKC was designed using the concept of evolutionary domain shuffling. It is one the major mechanisms leading to the formation of new proteins during the course of evolution^36^. As evolutionary domain shuffling is associated with emergence of novel biological function, synthetic domain shuffling can be applied to have proteins engineered with a novel cellular function. Chimeric PKC (462 amino acids) was designed by shuffling the PB1 and catalytic domain from PKC-ζ and PKC-α isoforms. The N terminal of chimeric PKC was tagged with 6XHIS.

### Homology modeling and MD simulation of the chimeric PKC

Since the chimeric PKC is a synthetically engineered protein, its 3D structure was modeled from well understood crystal structure of the ζ and α PKC isoforms, by homology modeling in Modeller (v 9.11)^37^. The template for the chimeric PKC was chosen by performing protein Basic Local Alignment Search Tool (pBLAST) against the Protein Data Bank (PDB) database using the amino acids sequence of the shuffled domains as the query sequence. Protein sequences with highest sequence similarity and identity were selected as templates for modelling chimeric PKC_ζα. BLASTp for 1WMH showed a query coverage of 98%, identity of 58% and e-value of 8e-32, while for 4RA4 the values were 92%, 99% and 0.0 respectively. 1WMH and 4RA4 are human homologues for the aPKC and cPKC repectively. There were other protein structures available in the PDB database with similar scores, but for homology modelling X-ray diffraction parameter of resolution was considered and the one with the best resolution was chosen. The PDB format for the crystal structures of the templates where retrieved from RCSB (Research Collaboratory for Structural Bioinformatics) PDB (www.rcsb.org).

The model that was built in Modeller had many outliers which were refined using the loop refinement or optimization protocol. The final model was then analyzed on ProSA-Web^38,39^ and RAMPAGE^40^ tool for model quality and to check for any errors pertaining to the *in silico* built 3D structure of the chimeric PKC_ζα. All the figures for the modeled chimeric PKC_ζα were generated in PyMOL Molecular Graphics System (v 1.7.4.5), Schrodinger, LLC^41^.

Stability analysis under physiological condition of the chimeric PKC_ζα homology model was performed by MD OPLS 2005 simulation in DESMOND 3.2 (D.E. Shaw Research) from Maestro 8.2^42^. Explicit transferable intermolecular potential with 3-points (TIP3P) water model was used in the orthorhombic box with a default 10 nm cutoff periodic boundary condition (PBC). Default long range and short range parameters were used along with a 2000 round of steepest descent minimization and Berendsen Normal Pressure temperature/Normal Volume Temperature (NPT/NVT) equilibration. After equilibration a production MD run was performed with a time step of 2 fs and for a time scale of 15 ns. All essential parameters such as potential energy, pressure, temperature were checked throughout the length of the MD simulation. The RMSD, RMSF and other parameters of the trajectories were also calculated using default Desmond event analysis protocols and minimum energy structures were exported from the trajectories for further analysis.

### Negative autoregulatory circuit design and its quasipotential landscape

The synthetic circuit with a LacI negative autoregulatory loop was designed in Tinker cell (www.tinkercell.com) and the chimeric PKC_ζα was coupled to it in such a way that its expression was under the transcriptional control of LacI repressor protein. Two ODEs depicting the negative autoregulatory structure of the circuit was written in Berkeley Madonna (v 8.3.18) (www.berkeleymadonna.com) and simulated using the Runge Kutta 4 (RK4) method. Time series data for 100 s was generated and analysed by Gene Regulatory Network Inference using Time Series (GRENITS) (v 1.24.0)^23^ and BoolNet (v 2.1.3)^43^ packages in Bioconductor’s R console. Circuit’s convergence, possible attractor states, the probability of Boolean network transitions and network wiring were created. Nullclines for the system of ODEs was also generated in Berkeley Madonna. The quasipotential landscape depicting the switching mechanism was inferred using the equation Vq = −((LacR) + (PKC))*DT derived from the Waddington’s epigenetic landscape^44^.

### Synthesis, expression and identification of the chimeric PKC

The amino acid sequence of the chimeric PKC_ζα protein was used as input for the synthesis by GeneArt, Invitrogen®. The sequence was reverse translated (1422 bp) and codon optimized for *E*. *coli* expression system. The synthesized nucleic acid was inserted into the pET151/D-Topo cloning vector. To confirm the presence of the chimeric PKC_ζα in the vector restriction digestion was done with EcoRI and HindIII. The sequence of the insert was also confirmed by sequencing. After insert confirmation the construct was transformed into competent BL21 (DE3) by heat shock treatment at 42 °C for 30 sec. These cells were plated on Luria Berttini (LB) agar plates containing ampicillin (selective marker) and incubated at 37 °C overnight. The transformed cells (ampicillin resistant) were selected and used for expression of the chimeric PKC_ζα. The expression of the protein was induced by 1 mM IPTG at 37 °C for 1 hour and continued till 3 hours. The expression of the chimeric PKC_ζα was checked on SDS-PAGE and analysed by LC-MS/MS housed at the NCCS, Proteomics facility.

### Western blot and confocal microscopy

Western blot and confocal microscopy was done to show the phosphorylation of IκKβ by the chimeric PKC_ζα. Peritoneal macrophages were isolated from Balb/c mice, post four days of injection of 3% thioglycolate into the peritoneal cavity of the mice^45^. Around 2 × 10^6^ cells/ml were plated and allowed to adhere to the culture vessel for 2–3 hours at 37 °C, 5% CO_2_ and humidity. The cells were cultured in Roswell Park Memorial Institute (RPMI) 1640 supplemented with 10% fetal bovine serum (FBS) The non-adherent cell were washed with warm phosphate buffered saline (PBS) and the cells were transfected with chimeric PKC_ζα construct using the Lipofectamine 3000® (Invitrogen®) following the manufacturer’s protocol. The transfected cells were induced with 1 mM IPTG for 24 hours. Cell lysate for western blotting was prepared by using cell lysis buffer, followed by quantification of protein by the bicinchoninic acid (BCA) method. Approximately 20 µg of protein was added per well for SDS-PAGE followed by wet transfer on to the nitrocellulose membrane. The membrane was blocked with 1% bovine serum albumin (BSA) for 1 hour at RT, followed by 16 hours incubation with the primary anti-mouse-phospho-IκKβ (Sigma®). The membrane after washing was probed with secondary antibody anti-rabbit conjugated to horse radish peroxidase (HRP) (BioRad®). The blot was developed using developer and the image was collected using the Amersham 600 Imager (General Electronics®). The densitometry analysis of the blots was done in ImageJ2 by normalizing with bActin^46^.

For confocal microscopy the transfected and induced peritoneal macrophages were fixed in 4% paraformaldehyde (PFA), followed by permeabilization in 0.1% Triton X. Blocking was done in 2% normal horse serum (NHS), after which cells were probed with primary anti-mouse-phospho-IκKβ at RT for 60 mins. After washing with phosphate buffered saline (PBS) secondary antibody conjugated to Alexa Flour 594 (a kind gift from Dr. Jomon Joseph, NCCS, Pune) was added to the cells and incubated for 30 mins at RT followed by washing with PBS. The slides were dried and mounted with VectaShield (Vector Laboratory ®) and observed under confocal microscope (Ziess LSM 510) housed at the NCCS Confocal facility.

### *In vitro* verification of the working of the synthetic circuit

The designed synthetic construct with a negative autoregulatory loop, was synthesized by GeneArt, Invitrogen®. The order of the parts used in the design of this construct is mentioned below and the sequence for the parts is given as S7. The parts were placed downstream of the CMV promoter in pcDNA 3.1(+). This construct was transfected into peritoneal macrophages by Lipofectamine 3000® reagent (Invitrogen) according to manufacturer’s protocol. After 24 hrs, transfection efficiency was calculated and was found to be around 20–30%. The working of the construct was ascertained, by induction using 1 mM IPTG for 24 hours and looking for GFP expression under fluorescence microscope (EVOS, Life Technologies®). Empty vector pcDNA 3.1(+) was also transfected in peritoneal macrophages, acting as a negative control.

### *In vitro* effect of cytokine gene expression as a result of action of chimeric PKC_ζα and miltefosine

The effectiveness of eliciting a pro-inflammatory response by the chimeric PKC_ζα in *L*. *major* infected macrophages, quantitative PCR analysis of cytokine gene expression and Griess’s assay for NO production was done. Peritoneal macrophages were collected from Balb/c mice as described previously and around 2 × 10^6^ cells/ml were plated in culture vessel. Eight treatment groups were designed; namely Infection (I), LPS, Empty Vector (EV), Chimeric PKC (CT), Chimeric PKC + Infection (CTI), Chimeric PKC + Miltefosine (CTM) and Chimeric PKC + Infection + Miltefosine (CTIM), which were compared to the untreated control (C) group.

For I, MI, CTI and CTIM groups, peritoneal macrophages were infected with stationary phase 2 × 10^7^ and 1 × 10^7^ *L*. *major* (MHOM/IL/67/JERICHO II) promastigotes at a cell to parasite ratio of 1:10 to 1:5 respectively. After 16 hours, of incubation with the parasite the cells were extensively washed with PBS to remove adherent, extracellular parasites. The infectivity index calculation was performed by counting the number of intracellular parasites after DAPI staining using EVOS florescence microscope (60× magnification).

For treatment CTM, CTIM group miltefosine was used at a concentration of 5 µM which is non-cytotoxic to peritoneal macrophages (ascertained by MTT assay data not shown). The transfected cells were induced with 1 mM IPTG. RNA samples were collected at time intervals of 0, 24, 48 and 72 hours from all the treatment and control groups by the standard Trizol (Sigma®) method. cDNA was prepared by reverse transcription using the High Capacity cDNA Reverse Transcriptase Kit®, Applied Biosystems using the standard cycling conditions. This cDNA was used as template for qPCR assay using the Taqman primer probe (FAM - 6-carboxyfluorescein) mix listed in S8. Standard cycling condition for qPCR on the StepOnePlus™ Real-Time PCR System was implemented.

### Statistical analysis and animal ethics

All the experiments were performed in triplicates and one way ANOVA with Tukey’s correction was employed for statistical significance. All the statistical calculation and graphs thereof were drawn in GraphPad Prism 5.

All mice were maintained in compliance with the Institutional Animal Ethics guidelines. All animal experiments were approved by the Institutional Animal Ethics Committee (IAEC) (7/GO/c/99/CPCSEA) of the National Centre for Cell Science (IAEC Project Number-EAF/2013/B-198 and EAF/2015/B-198(I).

## Electronic supplementary material

Supplementary information
