# Supplementary material for: Transcription Factor Target Gene Network governs the Logical Abstraction Analysis of the Synthetic Circuit in Leishmaniasis
Source: Sci Rep. 2018 Feb 22;8:3464. doi: 10.1038/s41598-018-21840-w (PMC5823942; doi:10.1038/s41598-018-21840-w)

Title: Transcription Factor Target Gene Network governs the Logical Abstraction Analysis of the Synthetic Circuit in Leishmaniasis

Authors: Milsee Mol[1], Dipali Kosey[1], Ramanamurthy Boppana[1] and Shailza Singh[1*]

Affiliations: [1]National Centre for Cell Science, NCCS Complex, Ganeshkhind, SP Pune University Campus, Pune 411007

Supporting information

Supplementary S1: TFTG network analysis

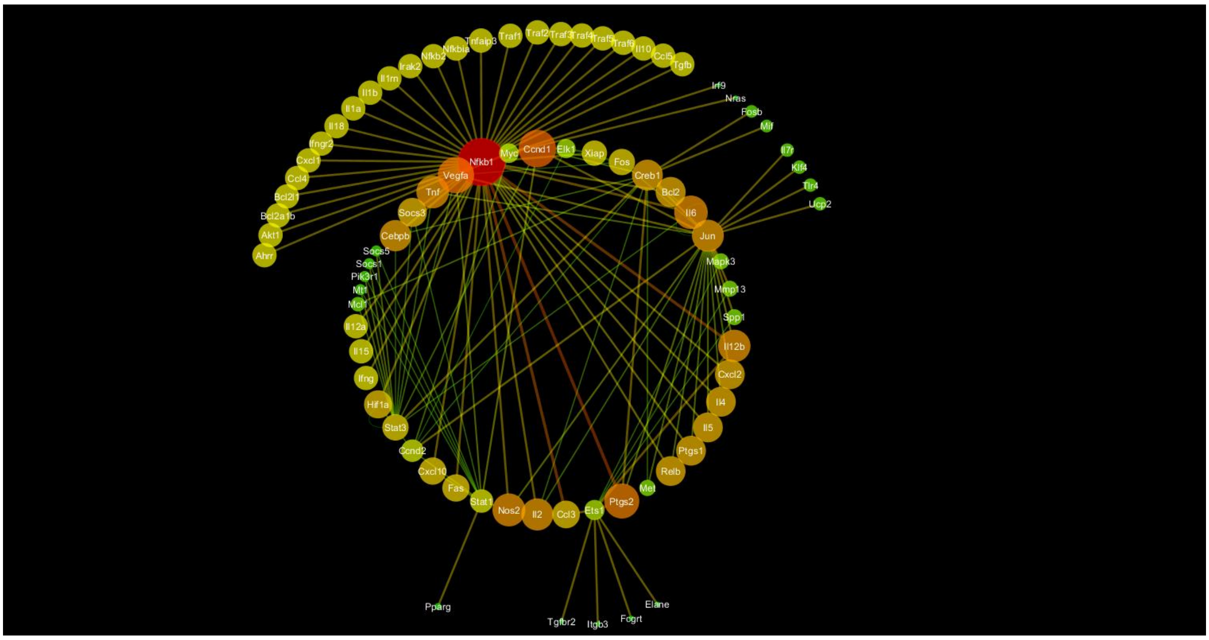

S1(a) TFTG network with NFκB (red) showing the maximum CC value in the network

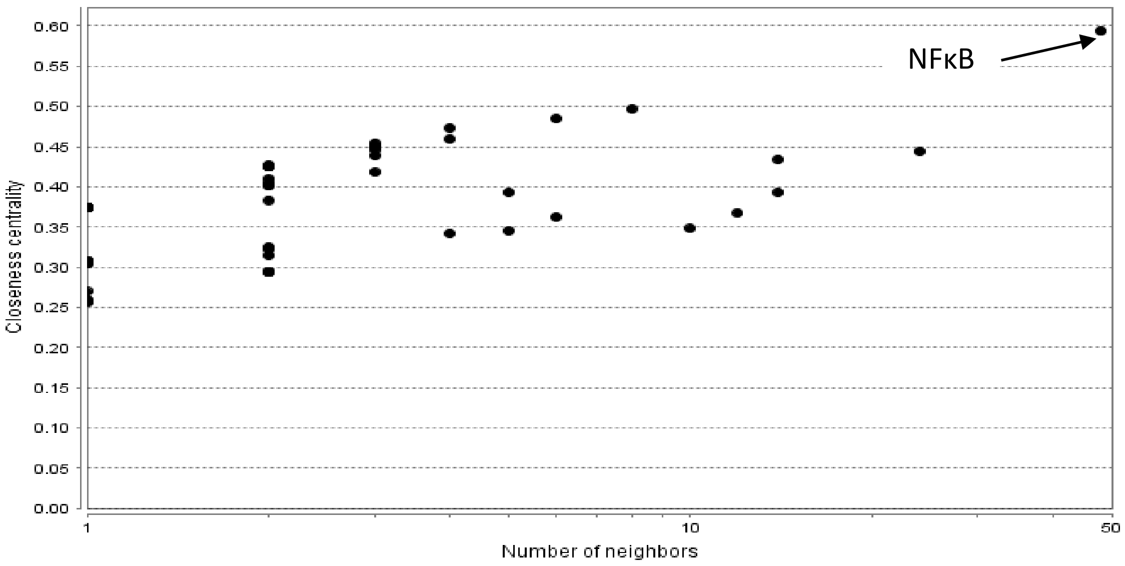

S1(b) CC values of all the nodes in the network and NFκB showing the highest CC value of 0.59

S1 (c) Whole TFTG network property

**Network Property**	**Value**
Number of Nodes	80
Clustering Coefficient	0.056
Network Diameter	6
Network Radius	3
Network Density	0.041
Characteristic Path length	2.772
Average no. of Neighbors	3.275

Supplementary S2: TFTG network evolvability analysis

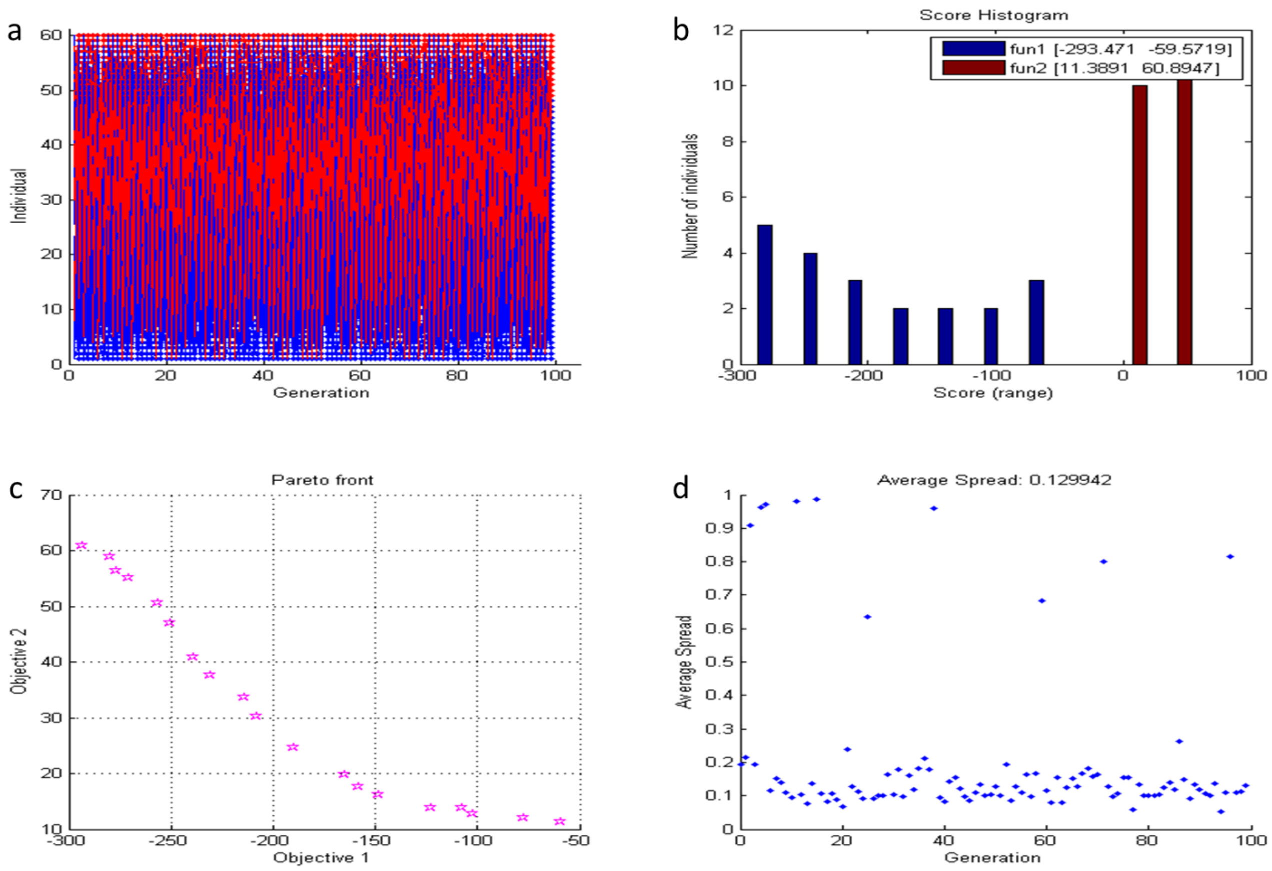

(a) NSGA-II algorithm preservs the 'elite' population through the GA run 100 generations
(b) The score histogram shows that f(1) is higher compared to f(2) and also the number of individuals i.e. solutions to the given objective function lie more in favour of f(1).
(c) The Pareto fronts for the opposing objective functions have 19 non-dominated solutions that are not discontinuous
(d) The average spread measure for the Pareto solutions is 0.129942

Supplementary S3: Chimeric PKC

S3 (a) The amino acid sequence used for domain shuffling, designing the chimeric PKC_ζα

<u>PBI Domain from PKCζ (Atypical PKC)</u>

N terminal domain: PB1 (16 – 98 aa)

>gi|84872200:16-98 protein kinase Cζ type isoform [*Mus musculus*]

VRLKAHYGGDILITSVDAMTTFKDLCEEVRDMCGLHQQHPLTLKWVDSEGDPCTVS
SQMELEEAFRLVCQGRDEVLIIHVFPS

<u>Catalytic Domain from PKCα (Conventional PKC)</u>

C terminal domain: Catalytic (290 – 668 aa)

>gi|164663791:290-668 protein kinase C α type isoform [*Mus musculus*]

IPEGDEEGNMELRQKFEKAKLGPAGNKVISPSEDRKQPSNNLDRVKLTDFNFLMVLG

KGSFGKVMLADRKGTEELYAIKILKKDVVIQDDDVECTMVEKRVLALLDKPPFLTQ

LHSCFQTVDRLYFVMEYVNGGDLMYHIQQVGKFKEPQAVFYAAEISIGLFFLHKRGI

IYRDLKLDNVMLDSEGHIKIADFGMCKEHMMDGVTTRTFCGTPDYIAPEIIAYQPYG

KSVDWWAYGVLLYEMLAGQPPFDGEDEDELFQSIMEHNVSYPKSLSKEAVSICKGL

MTKHPAKRLGCGPEGERDVREHAFFRRIDWEKLENREIQPPFKPKVCGKGAENFDKF

FTRGQPVLTPPDQLVIANIDQSDFEGFSYVNPQFVHPIL

S3 (b) the physiochemical properties of the chimeric PKC

Molecular weight: 52626.4 Da

Theoretical pI: 5.26

Amino acid composition:

**Aa**	**%**	**Aa**	**%**	**Aa**	**%**	**Aa**	**%**
Ala	4.5%	Leu	8.9%	Gln	4.3%	Ser	4.3%
Arg	4.3%	Lys	7.4%	Glu	8.0%	Thr	3.7%
Asn	2.6%	Met	3.5%	Gly	7.4%	Trp	0.9%
Asp	7.6%	Phe	5.8%	His	2.8%	Tyr	3.0%
Cys	2.4%	Pro	5.6%	Ile	5.6%	Val	7.4%

Instability index: The instability index (II) is computed to be 36.47, classifying the protein as stable.

Aliphatic index: 82.45

Grand average of hydropathicity (GRAVY): -0.316

Supplementary S4 : MD analysis of the chimeric PKC_ζα (pdf file)

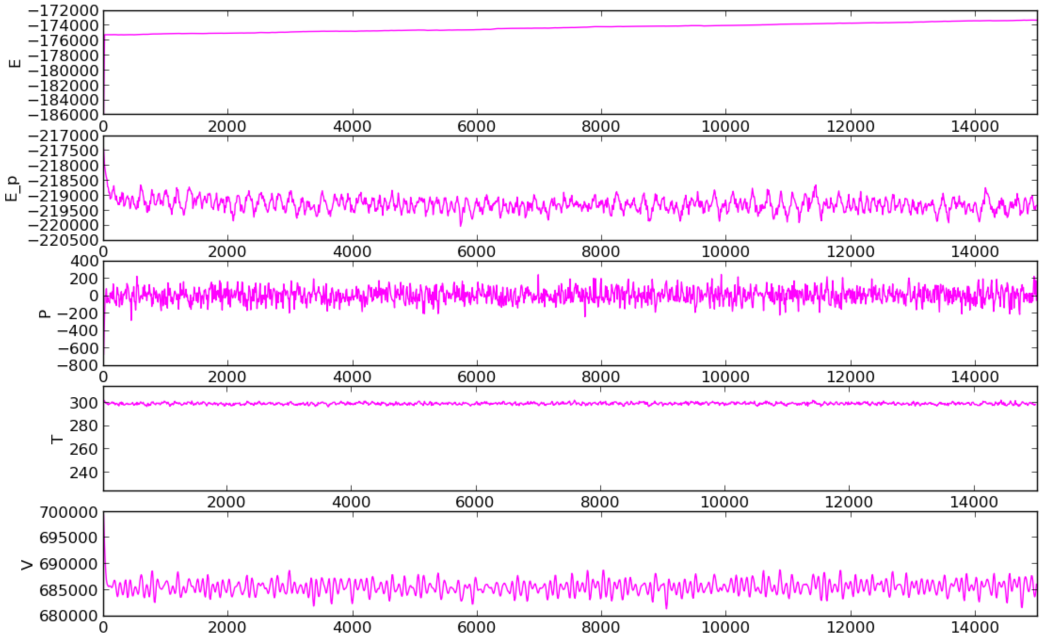

S4 (a): Physical parameters during the MD simulation were constant throughout the simulation time of 15ns

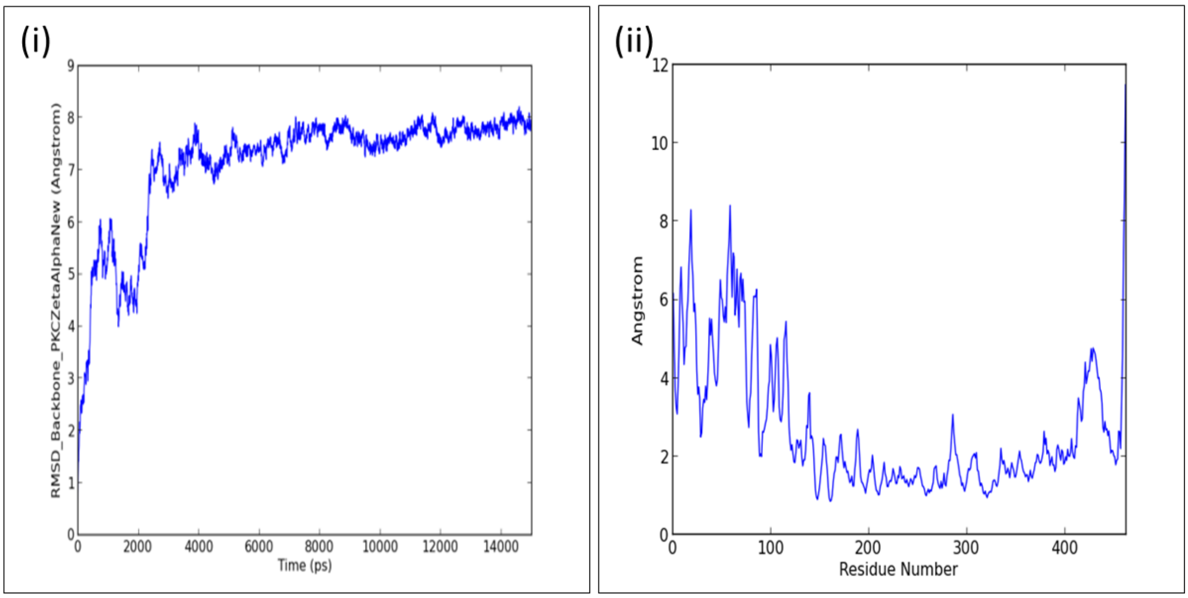

S4 (b): (i) RMSD plot of 15ns MD simulation for showing the mean fluctuation was at 8 Angstrom and stabilized after 10ns; (ii) RMSF plot of the 462 amino acids of the Chimeric PKC_ζα

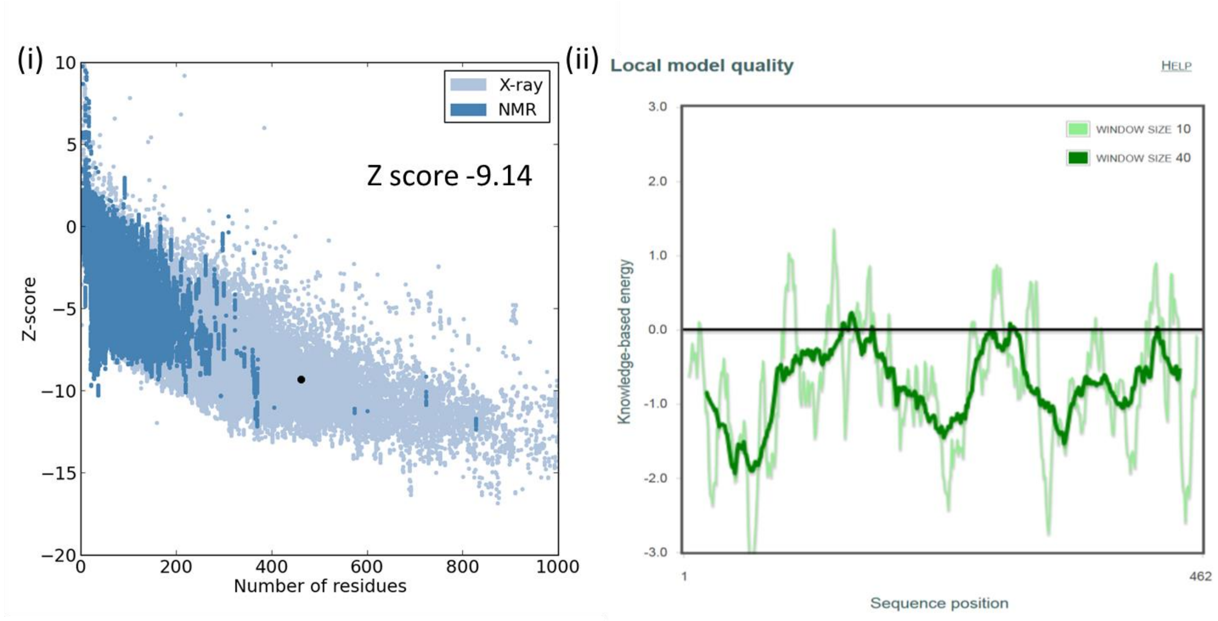

S4 (c): ProSA-Web Analysis of the chimeric PKC_ζα (i) the Z-score (ii) the energy plot for each amino acid

S4 (d) Rampage Analysis of chimeric PKC_ζα (Number of residues in favoured region (`98.0% expected): 444 (96.5%); Number of residues in allowed region (`2.0% expected) : 12 (2.6%); Number of residues in outlier region: 4 (0.9%)) (pdf file)

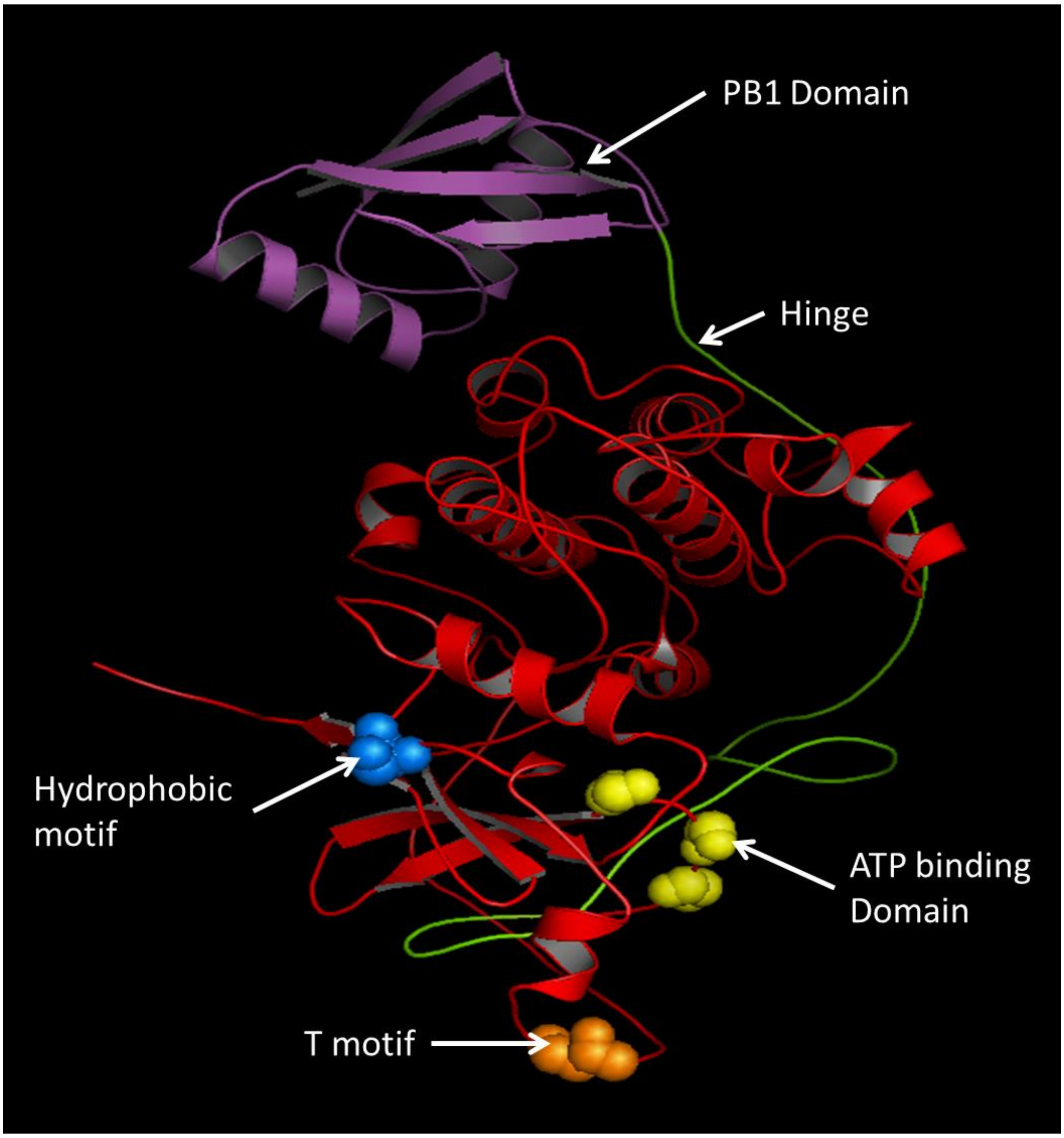

S4 (e) Final model of the chimeric PKC_ζα after MD showing the conserved regions; ATP binding domain (yellow): MVLG140KG142SFG145; T motif (orange): PVLT431PPDQL; Hydrophobic motif (blue): DFEGFS450YVNPQ; Purple is the PB1 domain and Red is the catalytic domain

Supplementary S5: Expression and identification of PKC_ζα

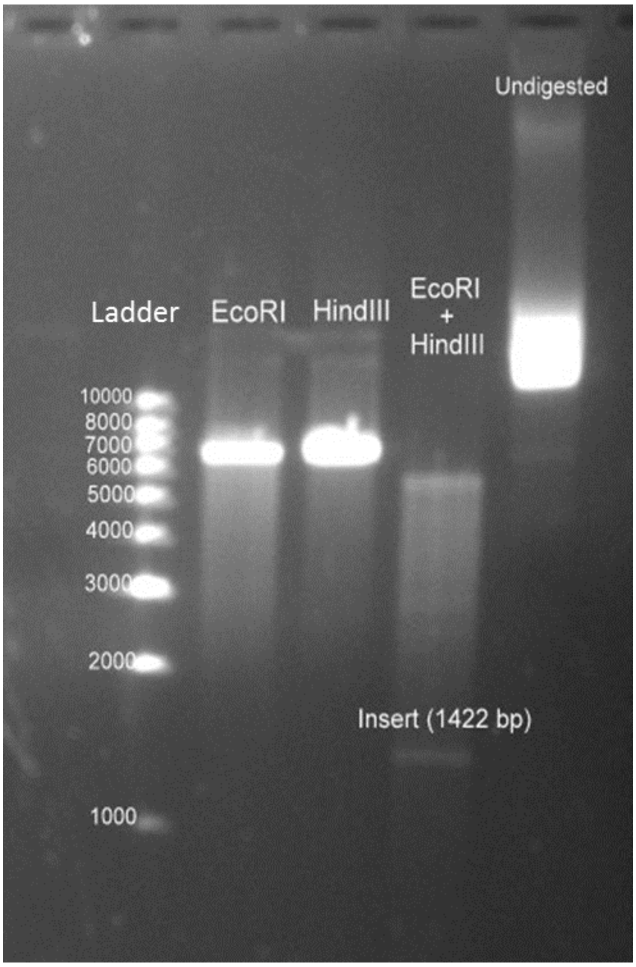

S5(a): Restriction digestion of vector pET-151/D-TOPO for expression of chimeric PKC_ζα

S5(b): MS analysis of the chimeric PKC_ζα (pdf file)

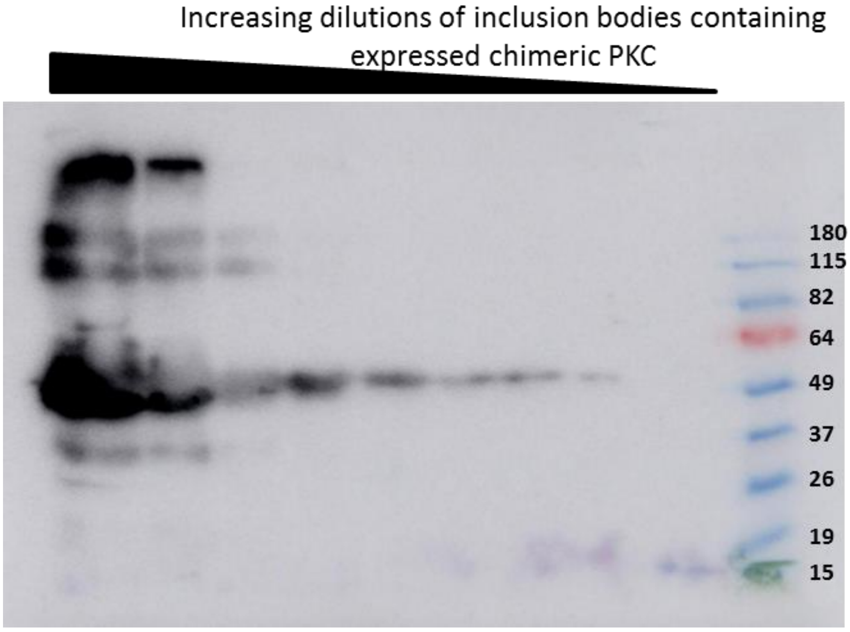

S5(c) anti-His probing of the His-tagged chimeric PKC

Supplementary S6: Synthetic Circuit

S6 (a): Equation for negative autoregulatory synthetic circuit simulated in Berkeley Madonna

```
METHOD RK4

STARTTIME = 0
STOPTIME=100
DT = 0.02

d/dt(PKC) = a1/(1+(LacR)^x - PKC
d/dt(LacR) = a2/(1+(IPTG)/c)^x – LacR+IPTG+pulse(1,0,24)

init LacR = 0
init PKC = 0
init Vq = 0

NEXT Vq = -((PKC^2)+(LacR)^2)*DT

a1 = 156.25; degradation rate of PKC
a2 = 15.6; degradation rate of LacR
c = 0.00035; dissociation constant of IPTG from LacR
x = 2 ; Hill coefficient
```

IPTG = 1; concentration in mM

DISPLAY LacR
DISPLAY PKC
DISPLAY Vq

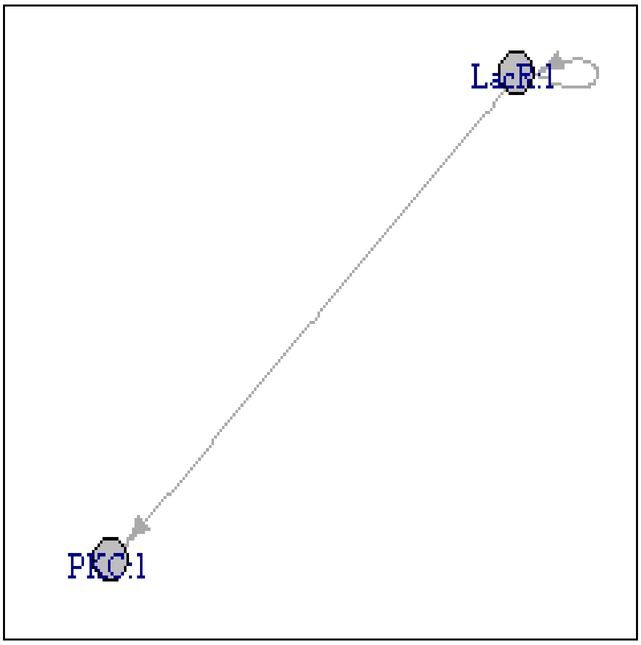

S6 (b): Circuit network wiring shows that the components are wired with respect to each other i.e. LacI R regulates itself and the expression of chimeric PKC

S6 (c): Convergence plots associated to the adequate convergence of the Markov Chain Monte Carlo (MCMC) (pdf file)

Supplementary S7: Sequence of the parts in the synthetic circuits

lacO2 operator (20 bp) part of BBa_K731500
GTGAGCGGATAACAATTCCC

LacI Repressor Protein (1086 bp) part of BBa_K731500
ATGAAACCAGTAACGTTATACGATGTCGCAGAGTATGCCGGTGTCTCTTATCAGA
CCGTTTCCCGCGTGGTGAACCAGGCCAGCCACGTTTCTGCGAAAACGCGGGAAA
AAGTGGAAGCGGCGATGGCGGAGCTGAATTACATTCCCAACCGCGTGGCACAAC
AACTGGCGGGCAAACAGTCGTTGCTGATTGGCGTTGCCACCTCCAGTCTGGCCCT
GCACGCGCCGTCGCAAATTGTCGCGGCGATTAAATCTCGCGCCGATCAACTGGGT
GCCAGCGTGGTGGTGTCGATGGTAGAACGAAGCGGCGTCGAAGCCTGTAAAGCG
GCGGTGCACAATCTTCTCGCGCAACGCGTCAGTGGGCTGATCATTAACTATCCGC
TGGATGACCAGGATGCCATTGCTGTGGAAGCTGCCTGCACTAATGTTCCGGCGTT
ATTTCTTGATGTCTCTGACCAGACACCCATCAACAGTATTATTTTCTCCCATGAAG
ACGGTACGCGACTGGGCGTGGAGCATCTGGTCGCATTGGGTCACCAGCAAATCG
CGCTGTTAGCGGGCCCATTAAGTTCTGTCTCGGCGCGTCTGCGTCTGGCTGGCTG

GCATAAATATCTCACTCGCAATCAAATTCAGCCGATAGCGGAACGGGAAGGCGA
CTGGAGTGCCATGTCCGGTTTTCAACAAACCATGCAAATGCTGAATGAGGGCATC
GTTCCCACTGCGATGCTGGTTGCCAACGATCAGATGGCGCTGGGCGCAATGCGC
GCCATTACCGAGTCCGGGCTGCGCGTTGGTGCGGATATCTCGGTAGTGGGATACG
ACGATACCGAAGACAGCTCATGTTATATCCCGCCGTTAACCACCATCAAACAGG
ATTTTCGCCTGCTGGGGCAAACCAGCGTGGACCGCTTGCTGCAACTCTCTCAGGG
CCAGGCGGTGAAGGGCAATCAGCTGTTGCCCGTCTCACTGGTGAAAAGAAAAAC
CACCCTGGCGCCCAATACGCAAACCGCCTCTCCCCGCGCGTTGGCCGATTCATTA
ATGCAGCTGGCACGACAGGTTTCCCGACTGGAAAGCGGGCAGTAATAA

Chimeric _PKC (1392 bp)
ATGGTGCGCCTGAAAGCGCATTATGGCGGCGATATTCTGATTACCAGCGTGGATG
CGATGACCACCTTTAAAGATCTGTGCGAAGAAGTGCGCGATATGTGCGGCCTGC
ATCAGCAGCATCCGCTGACCCTGAAATGGGTGGATAGCGAAGGCGATCCGTGCA
CCGTGAGCAGCCAGATGGAACTGGAAGAAGCGTTTCGCCTGGTGTGCCAGGGCC
GCGATGAAGTGCTGATTATTCATGTGTTTCCGAGCATTCCGGAAGGCGATGAAGA
AGGCAACATGGAACTGCGCCAGAAATTTGAAAAAGCGAAACTGGGCCCGGCGG
GCAACAAAGTGATTAGCCCGAGCGAAGATCGCAAACAGCCGAGCAACAACCTG
GATCGCGTGAAACTGACCGATTTTAACTTTCTGATGGTGCTGGGCAAAGGCAGCT
TTGGCAAAGTGATGCTGGCGGATCGCAAAGGCACCGAAGAACTGTATGCGATTA
AAATTCTGAAAAAAGATGTGGTGATTCAGGATGATGATGTGGAATGCACCATGG
TGGAAAAACGCGTGCTGGCGCTGCTGGATAAACCGCCGTTTCTGACCCAGCTGC
ATAGCTGCTTTCAGACCGTGGATCGCCTGTATTTTGTGATGGAATATGTGAACGG
CGGCGATCTGATGTATCATATTCAGCAGGTGGGCAAATTTAAAGAACCGCAGGC
GGTGTTTTATGCGGCGGAAATTAGCATTGGCCTGTTTTTTCTGCATAAACGCGGC
ATTATTTATCGCGATCTGAAACTGGATAACGTGATGCTGGATAGCGAAGGCCATA
TTAAAATTGCGGATTTTGGCATGTGCAAAGAACATATGATGGATGGCGTGACCAC
CCGCACCTTTTGCGGCACCCCGGATTATATTGCGCCGGAAATTATTGCGTATCAG
CCGTATGGCAAAAGCGTGGATTGGTGGGCGTATGGCGTGCTGCTGTATGAAATG
CTGGCGGGCCAGCCGCCGTTTGATGGCGAAGATGAAGATGAACTGTTTCAGAGC
ATTATGGAACATAACGTGAGCTATCCGAAAAGCCTGAGCAAAGAAGCGGTGAGC
ATTTGCAAAGGCCTGATGACCAAACATCCGGCGAAACGCCTGGGCTGCGGCCCG
GAAGGCGAACGCGATGTGCGCGAACATGCGTTTTTTCGCCGCATTGATTGGGAA
AAACTGGAAAACCGCGAAATTCAGCCGCCGTTTAAACCGAAAGTGTGCGGCAAA
GGCGCGGAAAACTTTGATAAATTTTTTACCCGCGGCCAGCCGGTGCTGACCCCGC
CGGATCAGCTGGTGATTGCGAACATTGATCAGAGCGATTTTGAAGGCTTTAGCTA
TGTGAACCCGCAGTTTGTGCATCCGATTCTGTAA

GFP (1257 bp) BBa_K194002
ATGTCTAAAGGTGAAGAATTATTCACTGGTGTTGTCCCAATTTTGGTTGAATTAG
ATGGTGATGTTAATGGTCACAAATTTTCTGTCTCCGGTGAAGGTGAAGGTGATGC
TACTTACGGTAAATTGACCTTAAAATTTATTTGTACTACTGGTAAATTGCCAGTTC
CATGGCCAACCTTAGTCACTACTTTCGGTTATGGTGTTCAATGTTTTGCGAGATAC
CCAGATCATATGAAACAACATGACTTTTTCAAGTCTGCCATGCCAGAAGGTTATG
TTCAAGAAAGAACTATTTTTTTCAAAGATGACGGTAACTACAAGACCAGAGCTG
AAGTCAAGTTTGAAGGTGATACCTTAGTTAATAGAATCGAATTAAAAGGTATTGA
TTTTAAAGAAGATGGTAACATTTTAGGTCACAAATTGGAATACAACTATAACTCT
CACAATGTTTACATCATGGCTGACAAACAAAAGAATGGTATCAAAGTTAACTTC
AAAATTAGACACAACATTGAAGATGGTTCTGTTCAATTAGCTGACCATTATCAAC
AAAATACTCCAATTGGTGATGGTCCAGTCTTGTTACCAGACAACCATTACTTATC
CACTCAATCTGCCTTATCCAAAGATCCAAACGAAAAGAGAGACCACATGGTCTT
GTTAGAATTTGTTACTGCTGCTGGTATTACCCATGGTATGGATGAATTGTACAAA

GCATCCAACTTGAACATTTCGAGAAAGCTTACCATATCAACCCCATCATGCTCTT
TCGAAAATTCAAATAGCACATCCATTCCTTCGCCCGCTTCCTCATCTCAAAGCCA
CACTCCAATGAGAAACATGAGCTCACTCTCTGATAACAGCGTTTTCAGCCGGAAT
ATGGAACAATCATCACCAATCACTCCAAGTATGTACCAATTTGGTCAGCAGCAGT
CAAACAGTATATGTGGTAGCACCGTTAGTGTGAATAGTCTGGTGAATACAAATA
ACAAACAAAGGATCTACGAACAAATCACGGGTCCTAACAGCAATAACGCAACCA
ATGATTATATTGATTTGCTAAACCTAAATGAGTCTAACAAGGAAAACCAAAATCC
CGCAACGGCGCATTACCTCAATGGGGGCCCACCCAAGACAAGCTTCATTAACCA
TGGAATGTTCCCCTCGCCAACTGGGACCATAAATAGCGGTAAATCTAGCAGTGCC
TCATCTTTAATTTCTTTTGGTATGGGCAATACCCAAGTAATATAGTAATAA

Terminator Sequence (41 bp) BBa_B0012
TCACACTGGCTCACCTTCGGGTGGGCCTTTCTGCGTTTATA

P2A Sequence (66bp) BBa_K1537016
GGTAGTGGAGCAACAAACTTCTCATTATTAAAGCAAGCAGGAGATGTGGAGGAA
AATCCTGGTCCA

T2A Sequence (63bp) BBa_K1537017
GGTAGTGGAGAGGGTAGAGGATCTCTTTTGACATGTGGAGATGTTGAAGAGAAC
CCTGGACCA

GSG linker sequence (9bp)
CCTAGGCCT

Supplementary S8: Primer probe mix used for the Cytokine qPCR Taqman Assay

Invitrogen® Assay ID	Gene
**Mm00434256_m1**	IL2
**Mm00445259_m1**	IL4
**Mm00439646_m1**	IL5
**Mm00446190_m1**	IL6
**Mm00439614_m1**	IL10
**Mm00434165_m1**	IL12α
**Mm00434174_m1**	IL12β
**Mm01168134_m1**	IFNγ
**Mm00443258_m1**	TNFα
**Mm01227699_m1**	TGFβ
**Mm00440502_m1**	iNOS
**Mm00607939_s1**	βActin

Supplementary S9: qPCR cytokine expression fold change of the treatment groups: Control (C), Infection (I), PKC Construct (CT), PKC Construct + Infection (CTI), PKC Construct + Infection + Miltefosine (CTIM)

	IL6	IL10	TGFb	TNFa	IL12A	IL12B	IFNG	iNOS
C:0	4E-04	3E-05	0.08	0.008	5E-04	2E-04	2E-05	6E-05
C:24	0.007	5E-04	0.018	0.105	0.021	0.021	2E-04	0.002
C:48	0.004	0.001	0.073	0.295	0.03	0.138	6E-04	0.19
C:72	0.018	1E-04	0.088	0.049	0.008	0.003	9E-05	0.047
I:0	0.001	3E-05	0.03	0.01	6E-04	0.001	3E-06	9E-05
I:24	4E-04	3E-05	0.025	0.006	2E-04	4E-04	3E-06	2E-05
I:48	6E-05	2E-05	0.004	3E-04	7E-06	3E-05	4E-06	6E-06
I:72	0.002	1E-04	0.108	0.077	0.003	0.003	8E-06	6E-04
CT:0	0.003	2E-04	0.007	0.03	6E-04	1E-04	8E-04	0.011
CT:24	0.001	1E-05	0.004	0.002	4E-05	1E-05	4E-05	1E-04
CT:48	0.001	2E-05	0.01	0.004	1E-04	2E-05	1E-05	8E-04
CT:72	0.001	2E-05	0.017	0.011	8E-05	1E-05	1E-05	0.001
CTI:0	0.008	3E-04	0.006	0.011	1E-04	2E-04	2E-04	0.002
CTI:24	0.003	3E-05	0.003	0.002	3E-05	1E-05	1E-05	5E-04
CTI:48	0.001	4E-05	0.005	0.003	1E-05	3E-05	1E-05	0.001
CTI:72	0.005	4E-05	0.011	0.004	2E-05	6E-05	4E-05	6E-04
CTIM:0	0.002	5E-04	0.007	0.008	9E-05	4E-05	1E-04	0.003
CTIM:24	0.003	1E-04	0.006	0.003	3E-05	3E-05	4E-05	0.002
CTIM:48	0.008	2E-04	0.011	0.006	6E-05	4E-05	1E-04	0.002
CTIM:72	0.008	2E-05	0.014	0.006	2E-05	4E-05	2E-05	0.003

Supplementary S10: NO analysis between Control (C), Lipopolysaccharide (LPS) and Empty Vector (EV)

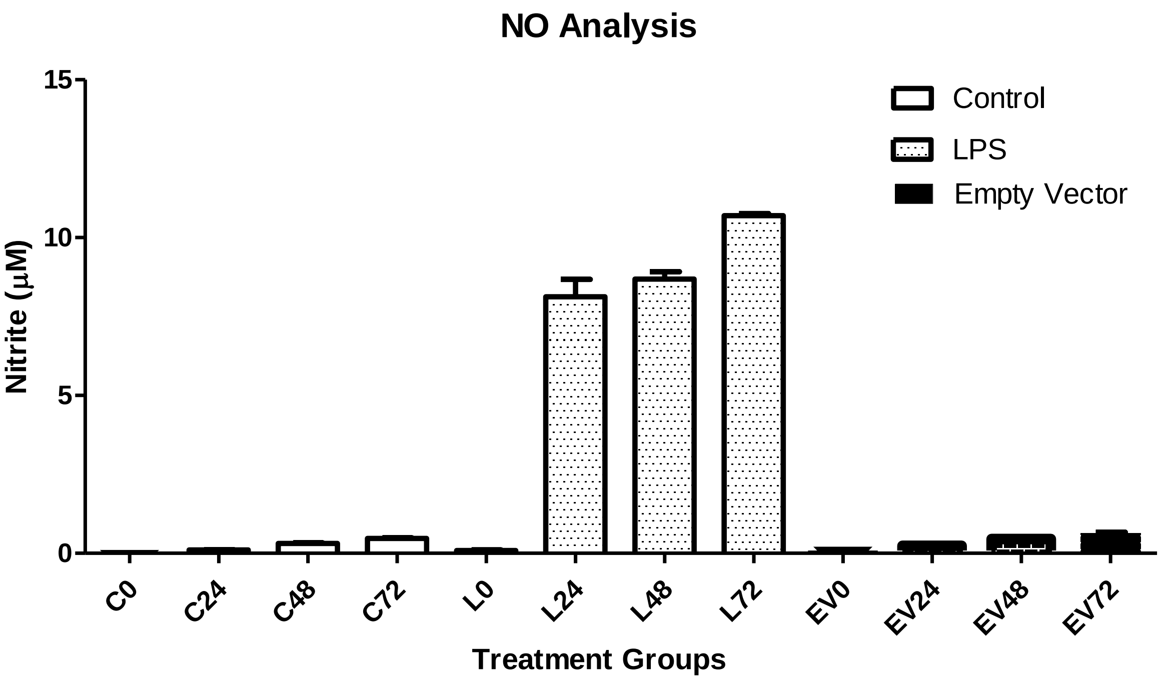

Supplement: Supplementary file 1 — Supplementary information [file 41598_2018_21840_MOESM1_ESM.pdf]
